# Molecular identification and mitogenomic characterization of cattle-associated *Trichophyton verrucosum*-related isolates from Kazakhstan

**DOI:** 10.3389/fvets.2026.1897150

**Published:** 2026-09-14

**Authors:** Mynbay Umitzhanov, Nazym Akimzhan, Altynai Arysbekova, Raikhan Boranbaeva, Begali Kanatov, Amangeldy Maulanov, Andrey Bogoyavlenskiy, Yergali Moldakhanov, Madina Alexyuk, Zhandos Abay, Ainur Nurpeisova, Markhabat Kassenov, Tatyana Titova, Abylai Sansyzbay, Nurbol Bakirov

**Affiliations:** 1 Kazakh National Agrarian Research University, Almaty, Kazakhstan; 2 Kazakh Scientific Research Veterinary Institute LLP, Almaty, Kazakhstan; 3 Research and Production Center for Microbiology and Virology, Almaty, Kazakhstan; 4 Ministry of Health of Russia, Bashkir State Medical University, Ufa, Russia

**Keywords:** cattle, dermatophytosis, ITS, Kazakhstan, mitochondrial genome, rabbit model, *Trichophyton verrucosum*, veterinary mycology

## Abstract

Dermatophytosis caused by zoophilic *Trichophyton* species remains an important infectious skin disease in cattle, but molecular data on veterinary dermatophytes from Central Asia are limited. This study characterized cattle-associated *Trichophyton verrucosum*-related isolates obtained from Kazakhstan using conventional mycology, experimental pathogenicity assessment, ITS-based phylogenetic analysis, and mitochondrial genome characterization. Twelve field *Trichophyton* isolates from six regions of Kazakhstan were examined. Cattle showed circumscribed alopecic and crusted skin lesions compatible with dermatophytosis, and fungal isolation was performed using affected hair and skin scales. In a rabbit scarified-skin model, isolates KZ_Tr1 and KZ_Tr2 from the Almaty region showed the highest lesion involvement among the tested field isolates, reaching 40, 60, and 80% at 3.0 × 10^6^, 5.0 × 10^6^, and 8.0 × 10^6^ microconidia/cm^3^, respectively. These two isolates were selected for molecular and mitogenomic analysis. ITS sequences of KZ_Tr1 and KZ_Tr2 clustered within a *T. verrucosum*-related lineage, although species-level interpretation was treated cautiously because the analysis was based on a single rDNA/ITS marker. Illumina sequencing enabled reconstruction of circular mitochondrial assemblies of 24,216 bp for KZ_Tr1 and 24,317 bp for KZ_Tr2. KZ_Tr2 showed stronger assembly quality metrics, including complete genome coverage, higher read depth, and fewer ambiguous nucleotide positions, and was therefore used as the main mitochondrial genome for detailed interpretation. Annotation of KZ_Tr2 identified 16 protein-coding genes, 24 tRNA genes, one small-subunit rRNA gene, and three tandem repeats. These findings provide the first molecular and mitochondrial genome data for cattle-associated *T. verrucosum*-related isolates from Kazakhstan and establish a regional baseline for future epidemiological, diagnostic, and comparative genomic studies of veterinary dermatophytes.

## 1 Introduction

Dermatophytosis is a contagious superficial fungal infection affecting keratinized tissues, including skin and hair, and remains an important problem in veterinary medicine because of its effects on animal health, farm management, and zoonotic risk. Dermatophytes are keratinophilic filamentous fungi that infect humans and animals, and several species are capable of transmission between animal hosts and humans (1, 2). In cattle, bovine ringworm is most commonly associated with *Trichophyton verrucosum*, although other dermatophytes, including members of the *Trichophyton mentagrophytes complex* and *Microsporum canis*, may occasionally be detected depending on host, environment, and management conditions (3). The disease is of practical importance because it can reduce animal productivity, affect the quality of animal-derived raw materials, and create occupational risks for farm workers and veterinarians (3–5).

Conventional diagnosis of bovine dermatophytosis relies on clinical signs, direct microscopy of affected hair and skin scales, and isolation of the fungus in pure culture. These methods remain essential for confirming viable dermatophytes and for obtaining isolates suitable for biological characterization. However, phenotypic identification may be complicated by slow growth, variation in colony morphology, and overlapping microscopic features among closely related dermatophytes (6, 7). Molecular approaches targeting the internal transcribed spacer region of ribosomal DNA are therefore widely used as complementary tools for dermatophyte identification and phylogenetic placement (6, 8). Recent veterinary studies have also emphasized the usefulness of molecular diagnostics for distinguishing *T. verrucosum*, *T. mentagrophytes* and *M. canis* in cattle-associated dermatophytosis (3, 9, 10).

Despite the veterinary importance of bovine dermatophytosis, molecular and genomic information on local cattle-associated dermatophyte isolates remains limited in many regions, including Central Asia. This gap is relevant because effective disease monitoring increasingly depends on reliable pathogen identification, strain documentation, and standardized surveillance approaches (11–13). In Kazakhstan, bovine dermatophytosis remains a persistent challenge for livestock production, but detailed sequence-based characterization of local Trichophyton isolates is still scarce (14, 15). Therefore, molecular documentation of experimentally selected local isolates may provide useful baseline information for future diagnostic, epidemiological, and veterinary biological studies.

Mitochondrial genomes provide additional markers for fungal characterization because they are compact, high-copy-number molecules encoding genes involved in oxidative phosphorylation, mitochondrial translation, ribosomal RNAs, and transfer RNAs (16, 17). In fungi, mitochondrial genome size and organization may vary due to introns, intergenic regions, repetitive elements, and accessory open reading frames. Comparative studies of dermatophyte mitogenomes have shown that mitochondrial data can contribute to understanding evolutionary relationships among dermatophyte genera and species (16, 18). Although nuclear rDNA/ITS sequences remain useful for initial identification, mitochondrial genome data can provide complementary information on genome organization and may improve molecular documentation of closely related or phenotypically similar dermatophyte isolates.

The present study aimed to characterize two selected based on lesion involvement in the rabbit model cattle-associated Trichophyton isolates from Kazakhstan. The isolates were selected based on comparative pathogenicity assessment in a rabbit model and were further analyzed using nuclear rDNA/ITS phylogeny and mitochondrial genome characterization. By combining conventional mycological examination, experimental pathogenicity assessment, rDNA/ITS sequencing, and mitogenome analysis, this study provides baseline molecular data for local *T. verrucosum*-related isolates recovered from cattle in Kazakhstan.

## 2 Materials and methods

### 2.1 Field isolates and mycological examination

A total of 12 field Trichophyton isolates were obtained from cattle with clinical signs compatible with dermatophytosis in six regions of Kazakhstan: Almaty, Kyzylorda, Turkestan, Mangystau, Akmola, and West Kazakhstan. The isolates were anonymized as KZ_Tr1–KZ_Tr12 for analysis and presentation. Pathological material consisted of affected hair and skin scales collected from lesion areas.

For direct microscopic examination, selected hair and scale fragments were placed on a glass slide in a drop of 20% sodium hydroxide solution used as a clearing agent. The preparation was gently heated and then mixed with a drop of warm sterile 50% aqueous glycerol solution before being covered with a coverslip and examined at ×10 and ×40 magnification. The presence of arthrospores and mycelial elements in hair shafts and skin scales was considered consistent with dermatophyte infection.

For fungal isolation, separate portions of affected hair and skin scales, not used for direct microscopy, were processed. To reduce surface contamination, the material intended for culture was briefly treated with 20% sodium hydroxide solution, washed with sterile distilled water, treated with 96% ethanol, and then washed again with sterile distilled water. After this surface-cleaning procedure, the material was inoculated onto wort agar and meat-peptone-glycerol agar supplemented with 2% glucose. Wort agar was selected based on established veterinary mycological practice and methodological recommendations indicating favorable growth of dermatophytes on this medium. Penicillin and streptomycin were added at 100–200 U/cm^3^ to suppress bacterial contamination. Cycloheximide was not added because contamination was controlled by sample preparation and antibiotic supplementation, and substantial growth of non-target fungi was not observed under the conditions used.

Cultures were incubated at 28 °C for up to 30 days and examined periodically. Initial colony growth on wort agar was observed on days 3–5, with well-developed colonies appearing during subsequent incubation. Fully developed cultures were obtained by approximately 18–21 days and were used for further biological assessment. Preliminary identification of recovered isolates was based on colony growth characteristics, macroscopic morphology, and microscopic features using standard mycological identification criteria.

### 2.2 Assessment of pathogenicity in rabbits and selection of isolates for sequencing

The pathogenicity of the 12 field Trichophyton isolates was evaluated using a rabbit scarified-skin infection model. Clinically healthy rabbits of both sexes, aged approximately 3–4 months and weighing 2.0–2.5 kg, were obtained from a rabbit facility with no history of dermatophytosis. Before the experiment, the animals were quarantined for one month and clinically examined to confirm the absence of visible skin lesions or signs compatible with fungal infection. Only animals without visible skin lesions and without clinical signs of disease were included in the experiment. Animals showing skin abnormalities, poor general condition, or signs of infectious disease during quarantine were excluded. Eligible rabbits were randomly allocated to experimental and control groups.

Each field isolate was tested at three inoculum concentrations: 3.0 × 10^6^, 5.0 × 10^6^, and 8.0 × 10^6^ microconidia/cm^3^. Three rabbits were used for each concentration; therefore, nine rabbits were used for each field isolate and 108 rabbits were used for the 12 field isolates. Two reference strains, *T. verrucosum F-02* and *T. mentagrophytes F-01,* were used as positive pathogenicity controls and were tested using the same dose scheme, with three rabbits per concentration. A negative-control group of three rabbits was inoculated with physiological saline. In total, 129 rabbits were included in the pathogenicity assessment. Animals were maintained under standard laboratory conditions with regular feeding and free access to water throughout the 15-day observation period.

For inoculation, hair was clipped from the dorsal region, and a 5 × 5 cm^2^ skin area was prepared. The skin surface was treated with 70% ethanol, and superficial scarifications were made using a sterile blade without inducing bleeding. Dermatophyte cultures grown for 18–21 days on wort agar were used for inoculum preparation. The fungal growth was suspended in sterile physiological saline, and the suspension was adjusted to the required concentration of microconidia. A 0.5 cm^3^ volume of the dermatophyte suspension was applied to the scarified area by gentle rubbing.

Lesion development was assessed on the inoculated 5 × 5 cm^2^ skin area during the 15-day observation period. The percentage values reported in this study indicate the visible proportion of the inoculated scarified area showing dermatophytosis-like lesions: complete involvement of the inoculated area was recorded as 100%, approximately half of the area as 50%, and lower values were assigned according to the visible extent of lesion involvement. In this study, “pathogenicity” refers to the ability of an isolate to induce dermatophytosis-like lesions in the rabbit model, whereas “lesion involvement percentage” refers to the extent of visible involvement of the inoculated skin area. The term “biological activity” was used to describe the combined evidence of reproducible lesion development and higher lesion involvement compared with other field isolates.

After comparative assessment of lesion development and dose response, the two field isolates showing the highest pathogenic activity were selected for molecular characterization and sequencing. These isolates were designated KZ_Tr1 and KZ_Tr2. All animal procedures were conducted in accordance with institutional animal welfare requirements and the principles of the 3Rs (19).

### 2.3 DNA extraction and quality assessment

Total DNA was extracted from pure fungal cultures of the two selected isolates using the PureLink Genomic DNA Mini Kit (Invitrogen, USA) according to the manufacturer’s instructions. DNA integrity was assessed by electrophoresis in 1% agarose gel followed by ethidium bromide staining. DNA concentration was measured using the Qubit dsDNA HS Assay Kit (Invitrogen, USA) on a Qubit 3.0 fluorometer. The A260/A280 ratio was measured using a Tecan instrument with a NanoQuant plate.

### 2.4 Library preparation, read processing, and genome assembly

Genomic DNA was extracted from fungal cultures using the PureLink Genomic DNA Mini Kit (Thermo Fisher Scientific, USA) according to the manufacturer’s instructions. DNA integrity was checked by electrophoresis in a 1% agarose gel.

Sequencing libraries were prepared from 1 ng of genomic DNA using the Nextera XT DNA Library Preparation Kit (Illumina, USA) according to the manufacturer’s protocol. Library purification and fragment size selection were performed using AMPure XP beads (Beckman Coulter, USA), followed by ethanol washing. Library quality and fragment size distribution were evaluated using an Agilent 2100 Bioanalyzer. Whole-genome sequencing was performed on the Illumina MiSeq platform using the MiSeq Reagent Kit v3, generating 300-bp paired-end reads.

Raw reads were assessed using FastQC and then trimmed and filtered using Trimmomatic v0.36 (20). Reads shorter than 50 bp after trimming were removed from downstream analysis. Filtered reads were assembled *de novo* using Geneious SPAdes based on SPAdes v3.12. To validate the mitochondrial assemblies, filtered reads were mapped back to the assembled mitochondrial contigs. Sequencing depth, genome coverage, read-mapping percentage, and consensus quality were calculated from the read-mapping results. Positions showing more than 10% nucleotide variation among aligned reads were designated as ambiguous nucleotide positions, and consensus sequences were manually inspected for polymorphisms and indels. Circular topology was supported by the presence of overlapping or highly similar terminal sequences and by read mapping back to the assembly.

### 2.5 Nuclear rDNA/ITS sequence analysis

For both selected isolates, the nuclear ribosomal DNA region comprising partial SSU rRNA, ITS1, 5.8S rRNA, ITS2, and partial LSU rRNA was obtained. Consensus sequences were compared with available sequences in public databases using BLASTn. Representative reference sequences of *Trichophyton* spp. and related dermatophytes were retrieved for phylogenetic analysis.

The final phylogenetic dataset included 26 nucleotide sequences: two Kazakh cattle isolates, representative reference sequences of *T. verrucosum, T. mentagrophytes, Trichophyton rubrum, Trichophyton tonsurans, Trichophyton benhamiae, Trichophyton erinacei, Trichophyton schoenleinii, Trichophyton violaceum, Arthroderma vanbreuseghemii* and *M. canis ATCC MYA-4605* as an outgroup. Sequences were aligned using MUSCLE in MEGA12 (21). To reduce the effect of unequal sequence lengths and terminal gaps, the alignment was trimmed to the shared nuclear rDNA/ITS core region. The final trimmed dataset contained 749 nucleotide positions. Phylogenetic analysis was performed in MEGA12 using the Neighbor-Joining method based on the p-distance model. Branch support was evaluated using 1,000 bootstrap replicates. Gaps and missing data were treated using pairwise deletion.

### 2.6 Mitochondrial genome annotation, visualization, and repeat analysis

Mitochondrial genome annotation was performed using MFannot (22). Transfer RNA genes predicted by MFannot were independently verified using tRNAscan-SE v2.0 in organellar mode with the mold and protozoan mitochondrial genetic code (23). Circular mitochondrial genome maps were generated using OGDRAW (24). Tandem repeat sequences were identified using Tandem Repeats Finder v4.09 with default parameters: match = 2, mismatch = 7, indel = 7, match probability = 80, indel probability = 10, minimum alignment score = 50, and maximum period size = 500.

KZ_Tr2 was selected as the main mitochondrial assembly for detailed annotation and interpretation because it showed complete genome coverage, higher sequencing depth, a higher proportion of mapped reads, and substantially fewer ambiguous nucleotide positions than KZ_Tr1. KZ_Tr1 was retained as a preliminary mitochondrial assembly and used only for cautious comparison.

### 2.7 Ethics statement

Animal experiments were conducted in accordance with institutional and national guidelines for veterinary research and laboratory animal welfare. The study protocol was reviewed and approved by the Ethics Committee for Animal Experiments of the Kazakh National Agrarian Research University (Protocol No. 8, April 01, 2023).

## 3 Results

### 3.1 Clinical presentation, direct microscopy, and fungal isolation

Cattle included in the study showed clinical signs compatible with dermatophytosis, including circumscribed alopecic skin lesions with scaling and crust formation. Lesions were mainly observed on the head and periocular areas of affected calves. Representative clinical lesions are shown in Supplementary Figure S2. Pathological material was collected from the active margins of affected skin areas and included hair and skin scales.

Direct microscopic examination of hair and scale fragments treated with 20% sodium hydroxide clearing solution revealed fungal elements consistent with dermatophyte infection. Microscopic findings included fungal structures associated with affected hair shafts and skin scales, supporting the preliminary diagnosis of dermatophytosis.

Fungal growth was obtained after inoculation of pathological material onto wort agar and meat-peptone-glycerol agar supplemented with 2% glucose. Initial colony development on wort agar was observed on days 3–5 of incubation at 28 °C. Colonies became clearly visible during subsequent incubation, and 18–21-day-old cultures were used for experimental pathogenicity assessment in rabbits. In total, 12 field Trichophyton isolates were included in the comparative analysis and were anonymized as KZ_Tr1–KZ_Tr12.

Macroscopic examination showed dermatophyte-like colonies on wort agar, while microscopic examination of fungal cultures revealed hyphal elements and chains of arthrospore-like cells consistent with the genus *Trichophyton* (Supplementary Figure S3). Based on clinical presentation, direct microscopy, cultural morphology, and subsequent molecular analysis, the isolates were regarded as cattle-associated *T. verrucosum-*related *Trichophyton* isolates.

### 3.2 Pathogenicity assessment in rabbits

The pathogenicity of the field *Trichophyton* isolates was evaluated in rabbits using a scarified skin infection model. The tested isolates originated from cattle in Almaty, Kyzylorda, Turkestan, Akmola, Mangystau, and West Kazakhstan regions. Experimental infection was performed by applying 0.5 cm^3^ of fungal culture onto a superficially scarified 5 × 5 cm^2^ dorsal skin area. Three inoculum concentrations were evaluated: 3.0 × 10^6^, 5.0 × 10^6^, and 8.0 × 10^6^ microconidia/cm^3^.

The field isolates showed dose-dependent differences in lesion development. Among all tested field isolates, the two isolates obtained from the Almaty region showed the highest lesion involvement. KZ_Tr1 and KZ_Tr2 produced lesion involvement values of 40, 60, and 80% at inoculum concentrations of 3.0 × 10^6^, 5.0 × 10^6^, and 8.0 × 10^6^ microconidia/cm^3^, respectively. In contrast, field isolates from Kyzylorda and Turkestan regions showed lower lesion involvement values of 20–40%, whereas isolates from Mangystau, Akmola, and West Kazakhstan regions showed values of 10–30% (Table 1).

**Table 1:** Dose-dependent lesion involvement of the sequenced Almaty isolates compared with reference strains in rabbits.

Isolate/strain	Origin	Rabbits per concentration	3.0 × 10^6^ microconidia/cm^3^, lesion involvement (%)	5.0 × 10^6^ microconidia/cm^3^, lesion involvement (%)	8.0 × 10^6^ microconidia/cm^3^, lesion involvement (%)	Interpretation
KZ_Tr1	Almaty region	3	40	60	80	Highest lesion involvement among field isolates
KZ_Tr2	Almaty region	3	40	60	80	Highest lesion involvement among field isolates
*T. verrucosum* F-02	Reference strain	3	100	100	100	Positive pathogenicity control
*T. mentagrophytes* F-01	Reference strain	3	100	100	100	Positive pathogenicity control
Physiological saline control	Negative control	3	0	0	0	No lesion development

Lesion involvement (%) indicates the visible proportion of the inoculated 5 × 5 cm^2^ scarified dorsal skin area showing dermatophytosis-like lesions during the observation period. Complete involvement of the inoculated area was recorded as 100%. The negative-control group was inoculated with physiological saline.

The reference strains *T. verrucosum F-02* and *T. mentagrophytes F-01* produced complete lesion involvement of the inoculated area at all tested concentrations, while the physiological saline control remained negative. Thus, KZ_Tr1 and KZ_Tr2 demonstrated the highest reproducible pathogenic activity among the tested field isolates and were selected for further molecular and mitochondrial genome characterization. The complete anonymized pathogenicity dataset is provided in Supplementary Table S1.

### 3.3 ITS/rDNA phylogenetic analysis

The rDNA/ITS sequences obtained from the two selected isolates were deposited in GenBank under accession numbers PZ441530 for KZ_Tr1 and PZ441531 for KZ_Tr2. Phylogenetic analysis was performed using representative ITS/rDNA sequences of Trichophyton species and *Microsporum canis* as an outgroup. Metadata for all reference sequences used in the analysis, including accession numbers, strain or isolate labels, host or source, and country or region when available, are provided in Supplementary Table S2.

In the neighbor-joining tree, KZ_Tr1 and KZ_Tr2 clustered within a group containing several reference sequences annotated in GenBank as *T. verrucosum*. This cluster was therefore referred to as a *T. verrucosum*-related lineage. In this study, the term “*T. verrucosum*-related” indicates that the isolates grouped phylogenetically with reference ITS/rDNA sequences assigned to *T. verrucosum*, while acknowledging that species-level identification based only on a single rDNA/ITS marker should be interpreted cautiously.

The two Kazakhstan isolates were separated from the main *T. mentagrophytes* cluster and from other dermatophyte species included in the tree. One sequence annotated as *T. verrucosum* BMU03357 was positioned outside the main *T. verrucosum*-related cluster and closer to the *T. mentagrophytes* group. This topology may reflect the limited discriminatory resolution of the ITS/rDNA region for some dermatophytes, differences in sequence length or quality among public GenBank records, or historical taxonomic annotation of deposited sequences. Therefore, the phylogenetic results support the placement of KZ_Tr1 and KZ_Tr2 within a *T. verrucosum*-related group but do not by themselves provide definitive species-level resolution (Figure 1).

**Figure 1:**
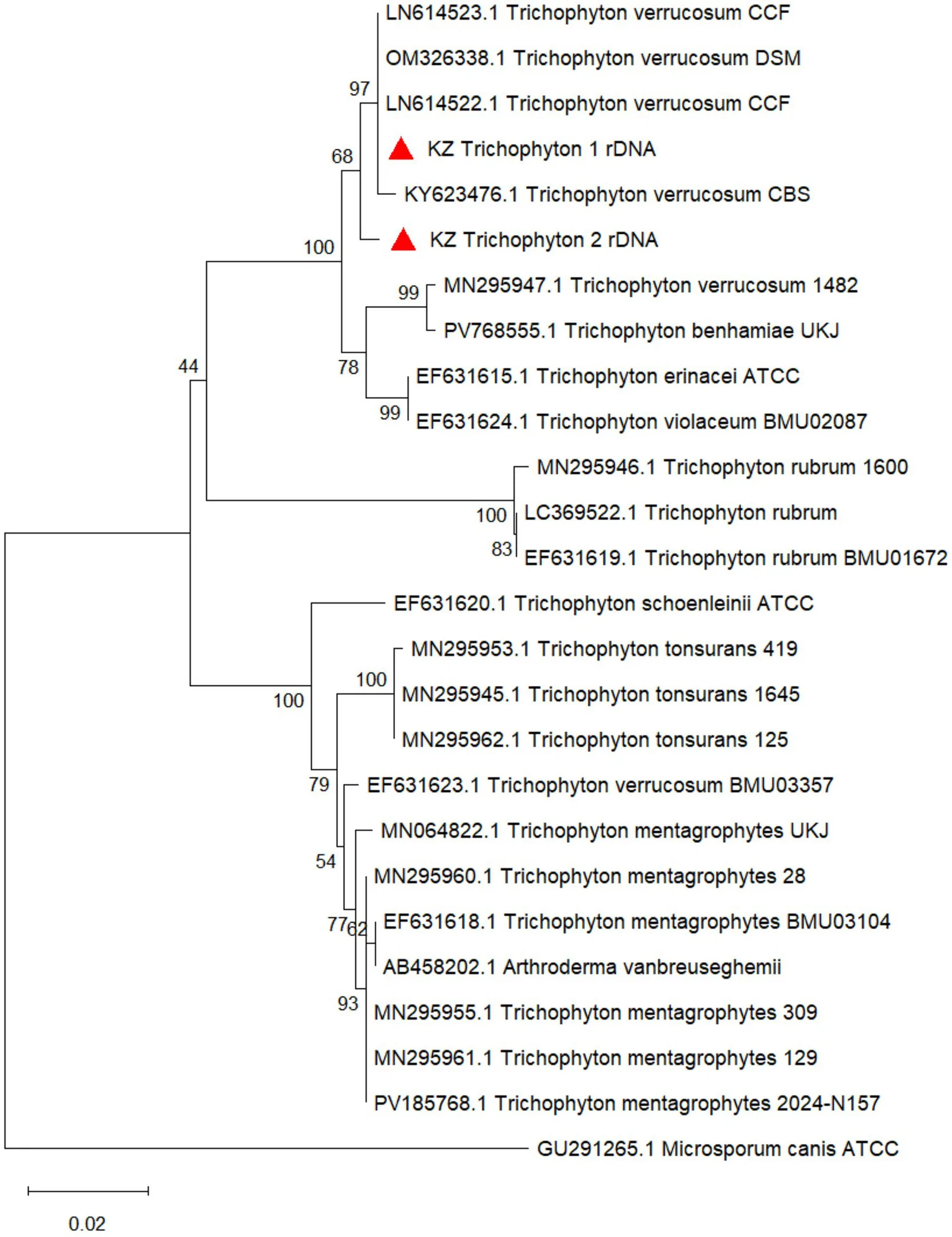
ITS/rDNA-based phylogenetic placement of the selected cattle-associated *Trichophyton* isolates KZ_Tr1 and KZ_Tr2. The neighbor-joining tree was constructed using ITS/rDNA sequences of representative *Trichophyton* species, with *Microsporum canis* used as the outgroup. The Kazakhstan isolates KZ_Tr1 and KZ_Tr2 are marked with red triangles. Bootstrap values are shown next to the branches. The scale bar indicates the number of substitutions per site. KZ_Tr1 and KZ_Tr2 clustered within a *T. verrucosum*-related lineage, while species-level interpretation was treated cautiously because the analysis was based on a single rDNA/ITS marker. Metadata for all sequences included in the tree are provided in Supplementary Table S2.

### 3.4 Mitochondrial genome assembly, quality assessment, and annotation

Illumina sequencing and *de novo* assembly enabled reconstruction of circular mitochondrial assemblies for the two selected cattle-associated *Trichophyton* isolates. The mitochondrial assembly of KZ_Tr1 was 24,216 bp in length, whereas the mitochondrial assembly of KZ_Tr2 was 24,317 bp. Both assemblies showed 100% mitochondrial genome coverage after read mapping back to the assembled contigs. The mean sequencing depth was 135× for KZ_Tr1 and 150× for KZ_Tr2, and the proportion of reads mapped back to the mitochondrial assemblies was 85 and 90%, respectively. Circular topology was supported by the presence of overlapping or highly similar terminal sequences and by read mapping back to the assembly.

Despite similar genome sizes and complete coverage, the two assemblies differed in consensus quality. KZ_Tr1 contained 1,279 ambiguous nucleotide positions, whereas KZ_Tr2 contained only 22 ambiguous positions. Therefore, KZ_Tr1 was treated as a preliminary mitochondrial assembly and was used only for cautious comparison. In contrast, KZ_Tr2 was selected as the main mitochondrial genome for detailed annotation and interpretation because it showed higher read depth, a higher read-mapping percentage, and substantially fewer ambiguous nucleotide positions.

Annotation of the KZ_Tr2 mitochondrial genome identified 16 protein-coding genes, *including cox1, cox2, cox3, cob, nad1, nad2, nad3, nad4, nad4L, nad5, nad6, atp6, atp8, atp9, rps3*, and *orf362*. In addition, one small-subunit rRNA gene and 24 tRNA genes were identified. The tRNA gene set predicted by MFannot was confirmed by tRNAscan-SE. No tRNA pseudogenes or tRNA introns were detected. Tandem Repeats Finder identified three tandem repeats in KZ_Tr2 and four tandem repeats in KZ_Tr1. The assembly-quality metrics and mitochondrial genome characteristics of both isolates are summarized in Table 2.

**Table 2:** Assembly quality metrics and mitochondrial genome characteristics of KZ_Tr1 and KZ_Tr2.

Feature	KZ_Tr1	KZ_Tr2
Assembly software	Geneious SPAdes/SPAdes v3.12	Geneious SPAdes/SPAdes v3.12
Assembly validation	Reads mapped back to mitochondrial assembly	Reads mapped back to mitochondrial assembly
Mitogenome size, bp	24,216	24,317
Genome coverage, %	100	100
Mean sequencing depth, ×	135	150
Phred quality threshold	*Q* > 30	*Q* > 30
Reads used for mitochondrial assembly/QC	4,500	5,000
Reads mapped to mitochondrial assembly, %	85	90
Genome topology	Circular	Circular
Evidence supporting circular topology	Overlapping/similar terminal sequences; read mapping back to assembly	Overlapping/similar terminal sequences; read mapping back to assembly
GC content, %	23.10	24.19
Ambiguous nucleotide positions	1,279	22
MFannot-predicted protein-coding genes	11	16
MFannot-predicted tRNA genes	16	24
tRNAscan-SE-confirmed tRNA genes	22	24
tRNA pseudogenes	0	0
tRNAs with introns	0	0
Tandem repeats detected by TRF	4	3
Interpretation	Preliminary mitochondrial assembly	Main mitochondrial assembly for detailed interpretation

KZ_Tr1 was considered preliminary because of the high number of ambiguous nucleotide positions, which may affect annotation reliability. KZ_Tr2 was used as the main mitochondrial genome for detailed interpretation because it had complete genome coverage, higher read depth, higher read-mapping percentage, and substantially fewer ambiguous nucleotide positions.

The preliminary circular mitochondrial genome map of KZ_Tr1 is provided as Supplementary Figure S1 (Figure 2).

**Figure 2:**
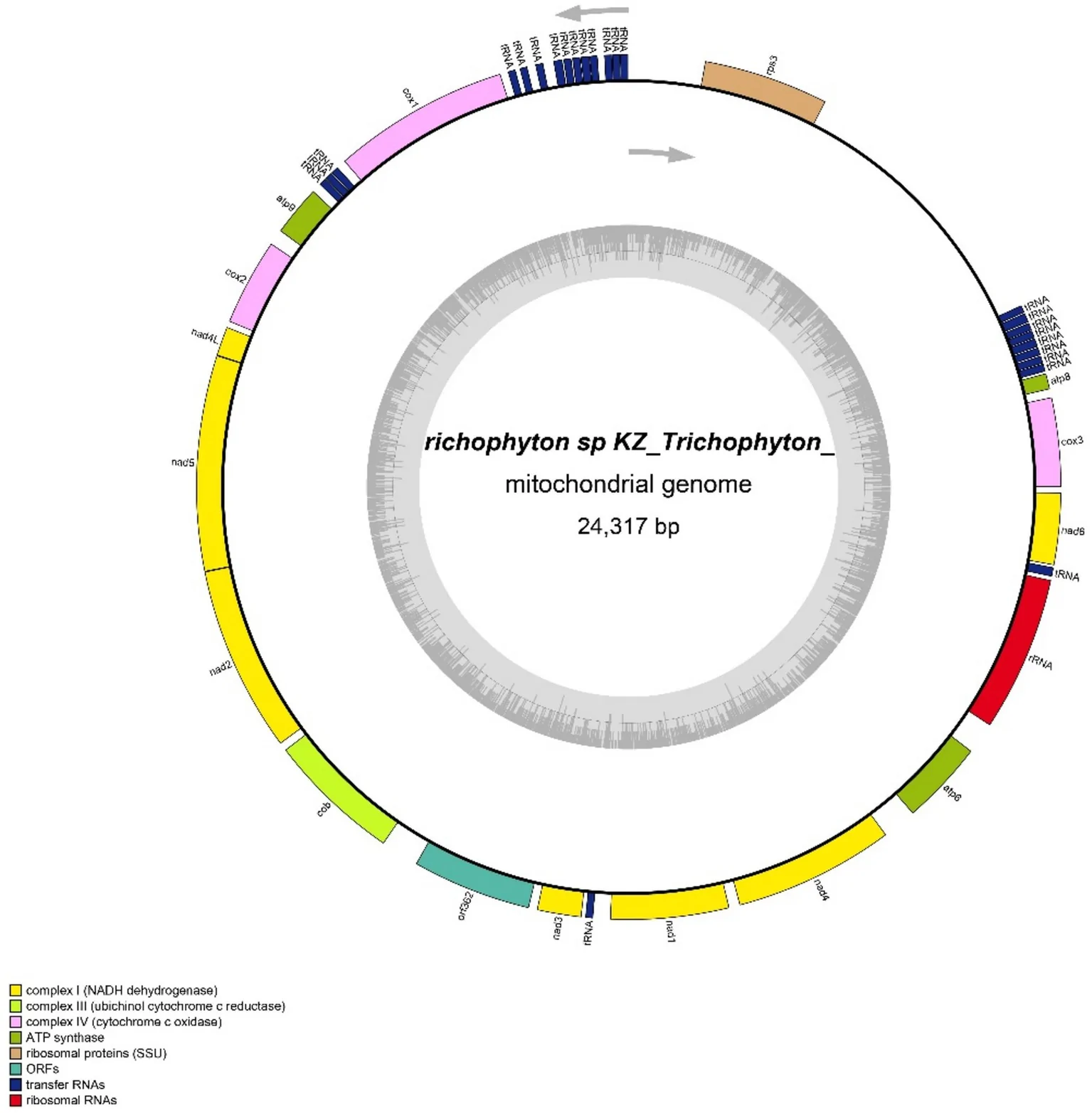
Circular mitochondrial genome map of the cattle-associated *Trichophyton verrucosum*-related isolate KZ_Tr2. The outer circle shows annotated mitochondrial genes, including protein-coding genes, tRNA genes, and the small-subunit rRNA gene. Genes shown outside and inside the circle indicate transcriptional orientation on opposite strands. The inner track represents GC-content variation across the mitochondrial genome. KZ_Tr2 was used for detailed mitochondrial genome visualization because it showed complete genome coverage, higher sequencing depth, and substantially fewer ambiguous nucleotide positions than KZ_Tr1.

### 3.5 tRNA gene confirmation and tandem repeat analysis

Independent tRNA prediction using tRNAscan-SE v2.0 in organellar mode confirmed 24 mitochondrial tRNA genes in KZ_Tr2. These tRNAs decoded the standard set of 20 amino acids. No tRNA pseudogenes, undetermined isotypes, or tRNA introns were detected. For KZ_Tr1, tRNAscan-SE predicted 22 internal-confirmed tRNA genes, with no pseudogenes, undetermined isotypes, or tRNA introns.

Tandem Repeats Finder identified three short tandem repeat regions in KZ_Tr2 at positions 4,645–4,682, 4,921–4,947, and 9,991–10,033. In KZ_Tr1, four short tandem repeat regions were identified at positions 4,645–4,682, 4,921–4,947, 6,005–6,050, and 6,053–6,081. The limited number of tandem repeats suggests compact mitochondrial genome organization in both isolates (Table 3).

**Table 3:** Tandem repeat regions detected in the mitochondrial genomes of KZ_Tr1 and KZ_Tr2.

Isolate	Position	Period size	Copy number	Consensus size	Matches	Indels	Score
KZ_Tr1	4,645–4,682	18	2.1	18	90%	9%	60
KZ_Tr1	4,921–4,947	13	2.1	13	100%	0%	54
KZ_Tr1	6,005–6,050	23	2.0	22	83%	4%	56
KZ_Tr1	6,053–6,081	15	1.9	15	100%	0%	58
KZ_Tr2	4,645–4,682	18	2.1	18	90%	9%	60
KZ_Tr2	4,921–4,947	13	2.1	13	100%	0%	54
KZ_Tr2	9,991–10,033	21	2.0	22	81%	9%	52

## 4 Discussion

This study provides molecular and mitochondrial genome characterization of two cattle-associated *Trichophyton verrucosum*-related isolates from Kazakhstan. Unlike studies focused only on diagnostic detection, the present work was based on culture-confirmed field isolates that were additionally evaluated in a rabbit scarified-skin model. This approach is relevant for veterinary mycology because viable and experimentally characterized local isolates are necessary for downstream studies of strain properties, epidemiology, and future applied veterinary research. Dermatophytosis remains an important fungal disease in cattle, and recent veterinary studies continue to highlight the need for reliable detection and differentiation of *T. verrucosum*, *T. mentagrophytes*, and *M. canis* in cattle-associated infections (3, 9, 10).

The pathogenicity assessment provided the experimental basis for selecting KZ_Tr1 and KZ_Tr2 for sequencing. In the revised manuscript, we avoided using “morbidity rate” as a numerical endpoint because the recorded percentages represented the visible proportion of the inoculated 5 × 5 cm^2^ scarified skin area showing dermatophytosis-like lesions rather than the proportion of diseased animals. Therefore, the endpoint is described as lesion involvement (%). Using this criterion, the two Almaty field isolates showed the highest lesion involvement among the tested local field isolates, reaching 40, 60, and 80% at increasing inoculum concentrations. Although the reference strains *T. verrucosum* F-02 and *T. mentagrophytes* F-01 produced complete lesion involvement under the conditions used, KZ_Tr1 and KZ_Tr2 were the most active among the local field isolates. This supported their selection for further molecular and mitochondrial genome characterization. The rabbit model should be interpreted as a comparative pathogenicity assay rather than a complete virulence study, because additional endpoints such as standardized lesion scoring, fungal burden, histopathology, or immunological profiling were not included.

The nuclear rDNA/ITS phylogenetic analysis placed KZ_Tr1 and KZ_Tr2 within a *T. verrucosum*-related lineage and separated them from the main *T. mentagrophytes* cluster. This placement is consistent with the cattle origin of the isolates, because *T. verrucosum* is one of the principal dermatophytes associated with bovine ringworm (3, 4). However, species-level interpretation based only on the ITS region should be made cautiously because closely related dermatophytes may show limited sequence divergence, and dermatophyte taxonomy has undergone substantial revision based on multilocus phylogenetic analyzes (6–8). For this reason, the isolates were conservatively described as *T. verrucosum*-related rather than assigned definitively to *T. verrucosum* on the basis of a single nuclear marker. The position of one public GenBank sequence annotated as *T. verrucosum* outside the main *T. verrucosum*-related cluster further supports cautious interpretation of single-locus phylogenies and may reflect limited ITS resolution, sequence-quality differences, or historical taxonomic annotation.

Although whole-genome Illumina sequencing was performed, the present manuscript focuses on mitochondrial genome characterization. This focus is justified because the mitochondrial assemblies were compact, circular, and supported by complete coverage after read mapping. In contrast, chromosome-level nuclear genome assembly and robust comparative nuclear genomics would require additional validation and preferably long-read sequencing, which was beyond the scope of the present study. Therefore, the current data should be interpreted as mitochondrial genome characterization rather than complete nuclear genome analysis.

The mitochondrial assembly of KZ_Tr2 represented the most reliable genomic result in this study. It was a circular molecule of 24,317 bp and displayed compact organization. MFannot annotation predicted 16 protein-coding genes, including mitochondrial genes associated with oxidative phosphorylation and mitochondrial translation. The annotation also included one small-subunit ribosomal RNA gene and 24 tRNA genes. The 24 tRNA genes predicted in KZ_Tr2 were independently confirmed using tRNAscan-SE v2.0 in organellar mode with the Mold and Protozoan mitochondrial genetic code. No tRNA pseudogenes or tRNA introns were detected. This agreement between MFannot and tRNAscan-SE supports the reliability of the KZ_Tr2 mitochondrial annotation (22, 23).

KZ_Tr1 had a similar mitochondrial genome size of 24,216 bp but contained a substantially higher number of ambiguous nucleotide positions. This affected automated annotation and resulted in fewer predicted protein-coding genes and fewer tRNA genes compared with KZ_Tr2. Therefore, the observed differences between KZ_Tr1 and KZ_Tr2 should not be interpreted as definitive evidence of biological gene loss or true structural divergence. Instead, they most likely reflect differences in consensus sequence quality and should be resolved by additional polishing or resequencing. For this reason, KZ_Tr2 was used as the main mitochondrial assembly for detailed interpretation, whereas KZ_Tr1 was treated as a preliminary mitochondrial assembly.

Tandem repeat analysis identified only a limited number of short tandem repeat regions in both mitochondrial assemblies. KZ_Tr2 contained three short tandem repeats, while KZ_Tr1 contained four. The low number of tandem repeats supports the compact organization of these mitogenomes and suggests that repeat expansion is not a major contributor to their size. This finding is relevant because fungal mitochondrial genome size variation is often associated with introns, intergenic regions, repetitive elements, and accessory open reading frames (17, 25). In the present study, however, the KZ_Tr2 mitogenome showed no evidence of extensive repeat-driven expansion. The tandem repeat screening performed with Tandem Repeats Finder therefore provides an additional structural description of these local isolates (26).

From a veterinary perspective, these findings provide baseline molecular data for cattle-associated *Trichophyton* isolates circulating in Kazakhstan. Such information may be useful for future surveillance, diagnostic assay development, and comparison of local isolates with reference strains. Broader cattle disease control studies have emphasized that surveillance programs benefit from standardized pathogen identification and documentation, even for diseases that differ in their degree of formal regulation between countries (11–13). In this context, deposition of the rDNA/ITS sequences in GenBank, provision of reference-sequence metadata, and characterization of mitochondrial genome structure contribute to improving the availability of regional dermatophyte data from Central Asia.

The study has several limitations. First, only two isolates were sequenced, and they were selected from a larger group of field isolates on the basis of lesion involvement in the rabbit model. Second, species-level identification was based mainly on ITS/rDNA phylogeny, and additional loci or whole-genome comparisons are needed for definitive taxonomic resolution. Third, the nuclear genome assemblies were not analyzed in detail, and no multilocus or whole-genome phylogeny was performed. Fourth, mitochondrial genome comparison was limited because publicly available, well-curated cattle-associated *T. verrucosum* mitogenomes remain scarce. Finally, KZ_Tr1 contained a relatively high number of ambiguous nucleotide positions and should be considered preliminary. Future studies should include a larger panel of field isolates, improved sequencing depth, long-read sequencing for complete nuclear and mitochondrial assemblies, and broader comparative analyses with international *Trichophyton* datasets. Such work will help determine whether mitochondrial genome features are stable across cattle-associated *T. verrucosum*-related isolates from Kazakhstan and whether they correlate with biological properties observed in experimental models.

Despite these limitations, the study provides new regional molecular and mitogenomic data for cattle-associated *T. verrucosum*-related isolates from Kazakhstan. The combination of conventional mycology, comparative pathogenicity assessment, ITS/rDNA phylogeny, and mitochondrial genome characterization establishes a useful baseline for future veterinary mycology, molecular surveillance, and comparative genomic investigations of dermatophytes in Central Asia.

## 5 Conclusion

This study provides the first molecular and mitogenomic characterization of cattle-associated *Trichophyton verrucosum*-related isolates from Kazakhstan. Among the evaluated field isolates, the two Almaty isolates KZ_Tr1 and KZ_Tr2 showed the highest lesion involvement in the rabbit scarified-skin model and were therefore selected for molecular analysis. ITS/rDNA phylogeny placed both isolates within a *T. verrucosum*-related lineage, although species-level interpretation should remain cautious because the analysis was based on a single nuclear marker. Mitochondrial genome analysis showed that KZ_Tr2 represents the main mitochondrial assembly suitable for detailed interpretation, with a compact circular mitogenome of 24,317 bp, whereas KZ_Tr1 should be regarded as a preliminary mitochondrial assembly because of its higher number of ambiguous nucleotide positions. Overall, these data establish a regional molecular baseline for cattle-associated dermatophytes in Kazakhstan and may support future veterinary surveillance, diagnostic development, comparative genomic studies, and further biological evaluation of local isolates.

## Data Availability

The original contributions presented in the study are publicly available. This data can be found here in GenBank under accession number: PZ896760, PZ441531 and PZ441530.

## References

[1] Cruciani D Papini M Broccatelli S Agnetti F Spina S Natalini Y . Presumptive zoonotic kerion by *Nannizzia gypsea*: case report. Front Vet Sci. (2021) 8:718766. doi: 10.3389/fvets.2021.718766, 34504889 PMC8421798

[2] Segal E Elad D. Human and zoonotic dermatophytoses: epidemiological aspects. Front Microbiol. (2021) 12:713532. doi: 10.3389/fmicb.2021.713532, 34421872 PMC8378940

[3] Wang Y Li Z Zuo Z Gu X Cai D Hu J . Establishment and application of multiplex PCR method for detection of *Trichophyton verrucosum*, *Microsporum canis*, and *Trichophyton mentagrophytes* from cattle. Front Vet Sci. (2025) 12:1546586. doi: 10.3389/fvets.2025.1546586, 40196810 PMC11973286

[4] Hameed K Riaz CF Nawaz MA Naqvi SMS Gräser Y Kupsch C . *Trichophyton verrucosum* infection in livestock in the Chitral district of Pakistan. J Infect Dev Ctries. (2017) 11:326–33. doi: 10.3855/jidc.7925 28459224

[5] Piorunek M Kubisiak-Rzepczyk H Trafas T Piorunek T. Superficial zoonotic mycoses in humans associated with cattle. Pathogens. (2024) 13:848. doi: 10.3390/pathogens13100848, 39452720 PMC11510394

[6] de Hoog GS Dukik K Monod M Packeu A Stubbe D Hendrickx M . Toward a novel multilocus phylogenetic taxonomy for the dermatophytes. Mycopathologia. (2017) 182:5–31. doi: 10.1007/s11046-016-0073-9, 27783317 PMC5283515

[7] Gnat S Łagowski D Dyląg M Ptaszyńska AA Nowakiewicz A. Modulation of ERG gene expression in fluconazole-resistant human and animal isolates of *Trichophyton verrucosum*. Braz J Microbiol. (2021) 52:2439–46. doi: 10.1007/s42770-021-00585-1, 34351602 PMC8578519

[8] Salehi Z Shams-Ghahfarokhi M Razzaghi-Abyaneh M. Molecular epidemiology, genetic diversity, and antifungal susceptibility of major pathogenic dermatophytes isolated from human dermatophytosis. Front Microbiol. (2021) 12:643509. doi: 10.3389/fmicb.2021.643509, 34149634 PMC8213211

[9] Łagowski D Gnat S Nowakiewicz A Trościańczyk A. Real-time PCR as an alternative technique for detection of dermatophytes in cattle herds. Animals. (2021) 11:1662. doi: 10.3390/ani11061662, 34199680 PMC8229919

[10] Spanamberg A Ravazzolo AP Araújo R Franceschi N Ferreiro L. Bovine ringworm—detection of *Trichophyton verrucosum* by SYBR-green real-time PCR. Med Mycol Case Rep. (2023) 39:34–7. doi: 10.1016/j.mmcr.2023.01.002, 36865767 PMC9971031

[11] Autio T Tuunainen E Nauholz H Pirkkalainen H London L Pelkonen S. Overview of control programs for cattle diseases in Finland. Front Vet Sci. (2021) 8:688936. doi: 10.3389/fvets.2021.688936, 34395573 PMC8361753

[12] Hodnik JJ Acinger-Rogić Ž Alishani M Autio T Balseiro A Berezowski J . Overview of cattle diseases listed under category C, D or E in the animal health law for which control programmes are in place within Europe. Front Vet Sci. (2021) 8:688078. doi: 10.3389/fvets.2021.688078, 34395571 PMC8361752

[13] Tamba M Pallante I Petrini S Feliziani F Iscaro C Arrigoni N . Overview of control programs for twenty-four infectious cattle diseases in Italy. Front Vet Sci. (2021) 8:665607. doi: 10.3389/fvets.2021.665607, 33981747 PMC8107282

[14] Umitzhanov M Abdiramanova B Abutalip A Bakirov N Sarimbekova AS. Comparative assessment of regulated methods and PCR in the diagnosis of trichophytosis in veterinary mycology. Open Vet J. (2023) 13:1614–22. doi: 10.5455/OVJ.2023.v13.i12.11, 38292703 PMC10824085

[15] Smagulova A Kukhar Y Glotova TI Glotov AG. Phylogenetic analysis of dermatophytes isolated from small domestic animals. Vet Sci Today. (2023) 12:259–64. doi: 10.29326/2304-196x-2023-12-3-259-264

[16] Wu P Yao T Ren Y Ye J Yuan Q Li Q . Evolutionary insights into two widespread ectomycorrhizal fungi (*Pisolithus*) from comparative analysis of mitochondrial genomes. Front Microbiol. (2021) 12:583129. doi: 10.3389/fmicb.2021.583129 PMC8287656 34290675

[17] Tang J Wang W Huang H Wang S. Insights into fungal mitochondrial genomes and inheritance based on current findings from yeast-like fungi. J Fungi. (2024) 10:430. doi: 10.3390/jof10070441 PMC11277600 39057326

[18] Zhan P Dukik K Li D Sun J Stielow JB Gerrits van den Ende B . Phylogeny of dermatophytes with genomic character evaluation of clinically distinct *Trichophyton rubrum* and *T. violaceum*. Stud Mycol. (2018) 89:153–75. doi: 10.1016/j.simyco.2018.02.004, 29910521 PMC6002342

[19] Mussoyev A Abutalip A Nurpeisova A Suchshikh V Makulbekov Y Yoo HS . Blackleg in cattle in Kazakhstan: regional epizootology, seasonal patterns, and molecular identification of the pathogen. Front Vet Sci. (2025) 12:1680881. doi: 10.3389/fvets.2025.1680881, 41133194 PMC12542868

[20] Bolger AM Lohse M Usadel B. Trimmomatic: a flexible trimmer for Illumina sequence data. Bioinformatics. (2014) 30:2114–20. doi: 10.1093/bioinformatics/btu170, 24695404 PMC4103590

[21] Kumar S Stecher G Suleski M Sanderford M Sharma S Tamura K. MEGA12: molecular evolutionary genetic analysis version 12 for adaptive and green computing. Mol Biol Evol. (2024) 41:msae263. doi: 10.1093/molbev/msae263, 39708372 PMC11683415

[22] Lang BF Beck N Prince S Sarrasin M Rioux P Burger G. Mitochondrial genome annotation with MFannot: a critical analysis of gene identification and gene model prediction. Front Plant Sci. (2023) 14:1222186. doi: 10.3389/fpls.2023.1222186, 37469769 PMC10352661

[23] Chan PP Lin BY Mak AJ Lowe TM. tRNAscan-SE 2.0: improved detection and functional classification of transfer RNA genes. Nucleic Acids Res. (2021) 49:9077–96. doi: 10.1093/nar/gkab688, 34417604 PMC8450103

[24] Greiner S Lehwark P Bock R. OrganellarGenomeDRAW (OGDRAW) version 1.3.1: expanded toolkit for graphical visualization of organellar genomes. Nucleic Acids Res. (2019) 47:W59–64. doi: 10.1093/nar/gkz238, 30949694 PMC6602502

[25] Kouvelis VN Hausner G. Editorial: mitochondrial genomes and mitochondrion related gene insights to fungal evolution. Front Microbiol. (2022) 13:897981. doi: 10.3389/fmicb.2022.897981, 35479620 PMC9036184

[26] Benson G. Tandem repeats finder: a program to analyze DNA sequences. Nucleic Acids Res. (1999) 27:573–80. doi: 10.1093/nar/27.2.573, 9862982 PMC148217

