# Supplementary material for: Molecular identification and mitogenomic characterization of cattle-associated Trichophyton verrucosum-related isolates from Kazakhstan
Source: Front Vet Sci. 2026 Sep 14;13:1897150. doi: 10.3389/fvets.2026.1897150 (PMC13618056; doi:10.3389/fvets.2026.1897150)

Supplementary Figure S1. Preliminary circular map of the mitochondrial genome of Trichophyton sp. KZ_Trichophyton_1.

The circular mitochondrial genome map was generated using MFannot annotation and visualized with OGDRAW. The mitochondrial genome is 24,216 bp in length. Because KZ_Trichophyton_1 contained a higher number of ambiguous nucleotide positions, several features remained incompletely annotated; therefore, this map should be interpreted as preliminary.

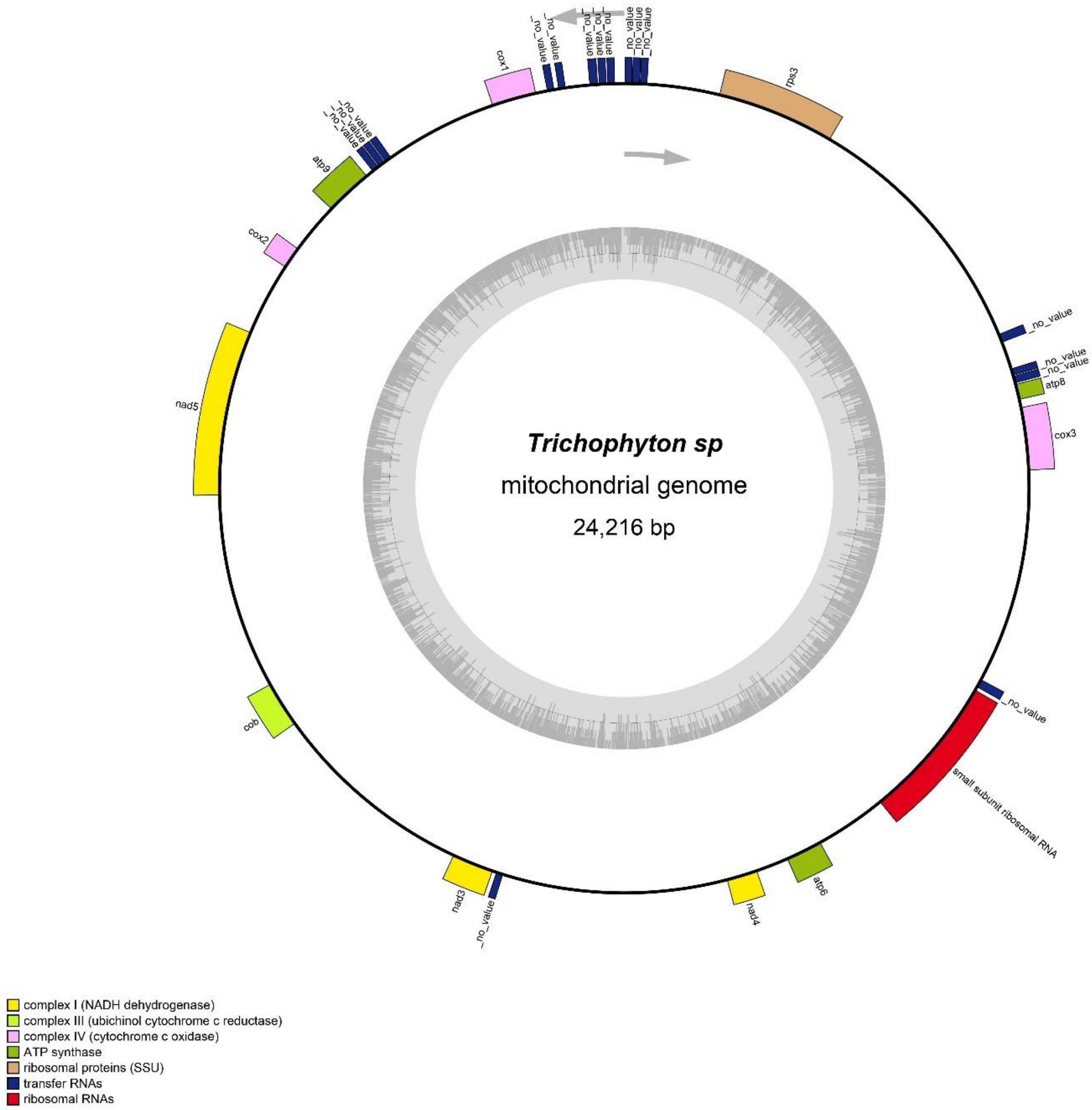

Supplement: Supplementary file 1 [file Image_1.pdf]
