# Supplementary material for: Molecular identification and mitogenomic characterization of cattle-associated Trichophyton verrucosum-related isolates from Kazakhstan
Source: Front Vet Sci. 2026 Sep 14;13:1897150. doi: 10.3389/fvets.2026.1897150 (PMC13618056; doi:10.3389/fvets.2026.1897150)
Supplement: Supplementary file 2 [file Image_2.pdf]

Supplementary Figure S2.
Macroscopic colony morphology and microscopic appearance of *Trichophyton verrucosum*

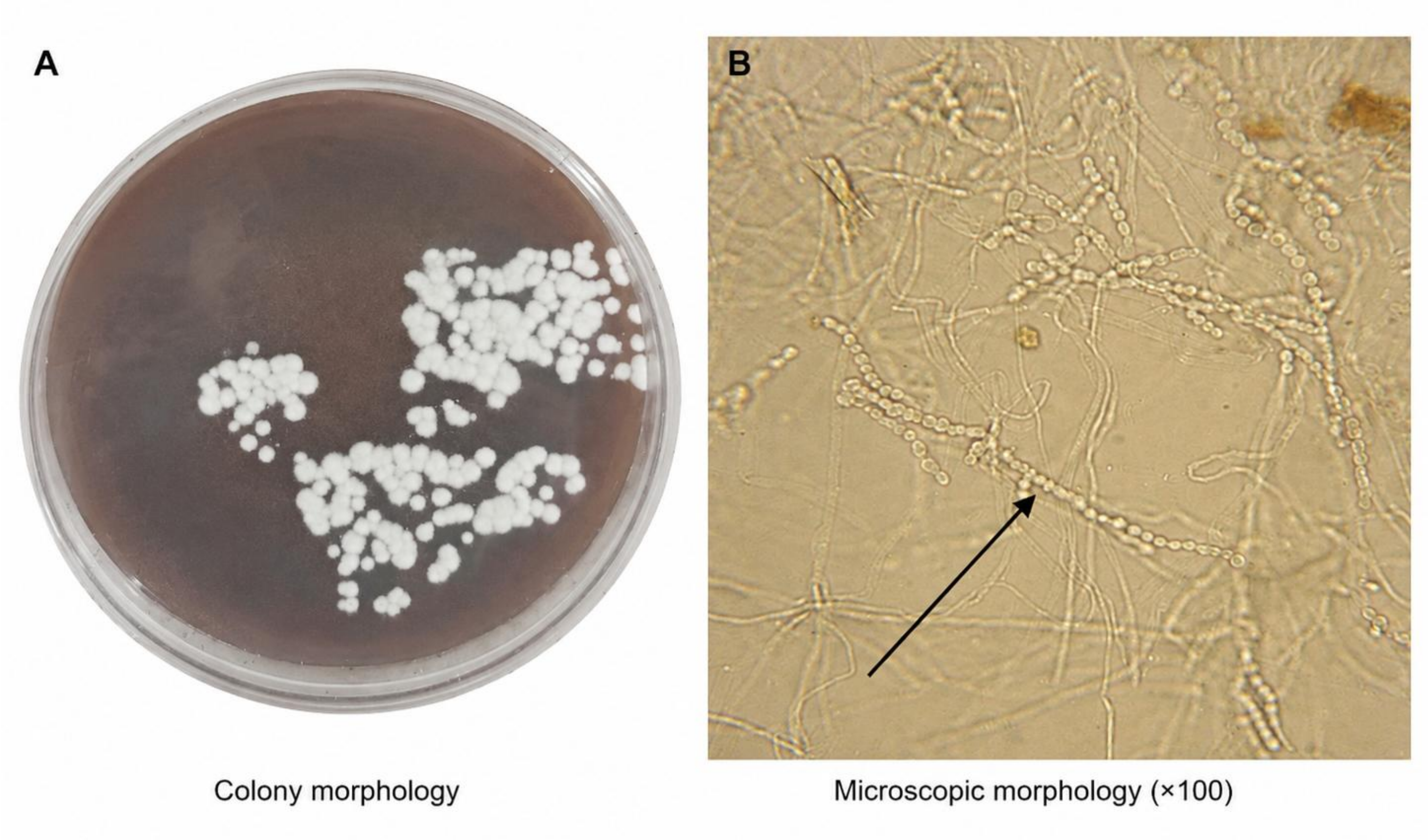

(A) Fungal colony grown on wort agar.
(B) Microscopic image (×100) showing hyphae and arthrospore-like elements (arrow).
