# Supplementary material for: Molecular identification and mitogenomic characterization of cattle-associated Trichophyton verrucosum-related isolates from Kazakhstan
Source: Front Vet Sci. 2026 Sep 14;13:1897150. doi: 10.3389/fvets.2026.1897150 (PMC13618056; doi:10.3389/fvets.2026.1897150)
Supplement: Supplementary file 3 [file Image_3.pdf]

Supplementary Figure S3.
Clinical dermatophytosis-like skin lesions in affected calves.

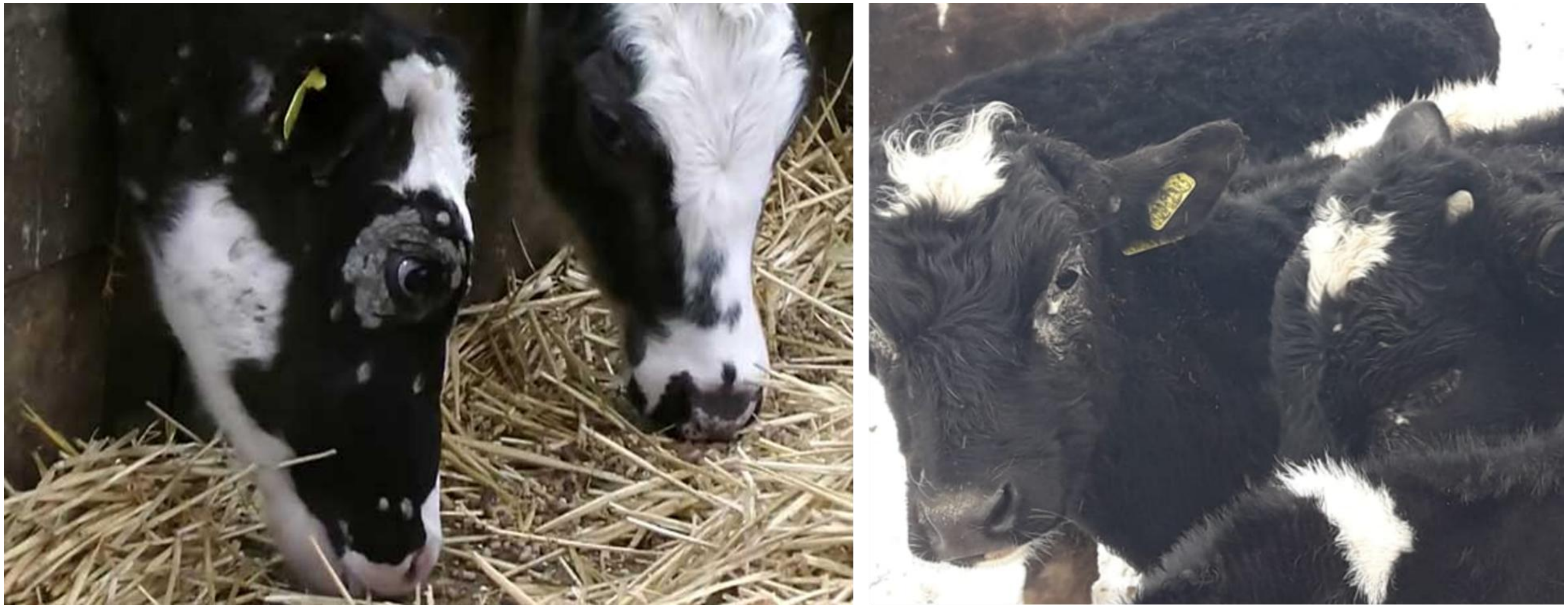

Representative calves showing circumscribed alopecic and crusted skin lesions compatible with cattle-associated dermatophytosis. The images illustrate the clinical material from which pathological samples were collected for mycological examination and isolation of field Trichophyton isolates.
