# Supplementary material for: Molecular identification and mitogenomic characterization of cattle-associated Trichophyton verrucosum-related isolates from Kazakhstan
Source: Front Vet Sci. 2026 Sep 14;13:1897150. doi: 10.3389/fvets.2026.1897150 (PMC13618056; doi:10.3389/fvets.2026.1897150)
Supplement: Supplementary file 4 [file Table_1.docx]

**Supplementary Table S1. Pathogenicity and virulence of anonymized field Trichophyton isolates and reference strains in rabbits.**

| **Isolate/strain code** | **Region of origin** | **Animal model** | **Inoculum volume** | **3.0 × 10^6^ microconidia/cm^3^, diseased/non-diseased** | **Morbidity at 3.0 × 10^6^, %** | **5.0 × 10^6^ microconidia/cm^3^, diseased/non-diseased** | **Morbidity at 5.0 × 10^6^, %** | **8.0 × 10^6^ microconidia/cm^3^, diseased/non-diseased** | **Morbidity at 8.0 × 10^6^, %** | **Selected for sequencing** | **Interpretation** |
| --- | --- | --- | --- | --- | --- | --- | --- | --- | --- | --- | --- |
| KZ_Tr1 | Almaty region | Rabbit | 0.5 cm^3^ | 4/6 | 40 | 6/4 | 60 | 8/2 | 80 | Yes | Highest pathogenicity among field isolates |
| KZ_Tr2 | Almaty region | Rabbit | 0.5 cm^3^ | 4/6 | 40 | 6/4 | 60 | 8/2 | 80 | Yes | Highest pathogenicity among field isolates |
| KZ_Tr3 | Kyzylorda region | Rabbit | 0.5 cm^3^ | NT | NT | 2/8 | 20 | 4/6 | 40 | No | Moderate pathogenicity |
| KZ_Tr4 | Kyzylorda region | Rabbit | 0.5 cm^3^ | NT | NT | 2/8 | 20 | 4/6 | 40 | No | Moderate pathogenicity |
| KZ_Tr5 | Turkestan region | Rabbit | 0.5 cm^3^ | NT | NT | 2/8 | 20 | 4/6 | 40 | No | Moderate pathogenicity |
| KZ_Tr6 | Turkestan region | Rabbit | 0.5 cm^3^ | NT | NT | 2/8 | 20 | 4/6 | 40 | No | Moderate pathogenicity |
| KZ_Tr7 | Mangystau region | Rabbit | 0.5 cm^3^ | NT | NT | 1/9 | 10 | 3/7 | 30 | No | Low pathogenicity |
| KZ_Tr8 | Mangystau region | Rabbit | 0.5 cm^3^ | NT | NT | 1/9 | 10 | 3/7 | 30 | No | Low pathogenicity |
| KZ_Tr9 | Akmola region | Rabbit | 0.5 cm^3^ | NT | NT | 1/9 | 10 | 3/7 | 30 | No | Low pathogenicity |
| KZ_Tr10 | Akmola region | Rabbit | 0.5 cm^3^ | NT | NT | 1/9 | 10 | 3/7 | 30 | No | Low pathogenicity |
| KZ_Tr11 | West Kazakhstan region | Rabbit | 0.5 cm^3^ | NT | NT | 1/9 | 10 | 3/7 | 30 | No | Low pathogenicity |
| KZ_Tr12 | West Kazakhstan region | Rabbit | 0.5 cm^3^ | NT | NT | 1/9 | 10 | 3/7 | 30 | No | Low pathogenicity |
| Negative control | Not applicable | Rabbit | 0.5 cm^3^ | 0/5 | 0 | 0/5 | 0 | 0/5 | 0 | No | No lesion development |
| *T. verrucosum* F-02 | Reference strain | Rabbit | 0.5 cm^3^ | See note | 100 | See note | 100 | See note | 100 | No | Positive virulence control |
| *T. mentagrophytes* F-01 | Reference strain | Rabbit | 0.5 cm^3^ | See note | 100 | See note | 100 | See note | 100 | No | Positive virulence control |

**Notes:** KZ_Tr1–KZ_Tr12 are anonymized field-isolate codes assigned to avoid disclosure of farm names.

NT, not tested. For field isolates, each inoculum group included 10 rabbits per dose. Experimental infection was performed by applying 0.5 cm^3^ of fungal culture onto scarified dorsal skin. KZ_Tr1 and KZ_Tr2 were selected for sequencing because they showed the highest morbidity among field isolates, reaching 80% at 8.0 × 10^6^ microconidia/cm^3^.
