# Supplementary material for: Molecular identification and mitogenomic characterization of cattle-associated Trichophyton verrucosum-related isolates from Kazakhstan
Source: Front Vet Sci. 2026 Sep 14;13:1897150. doi: 10.3389/fvets.2026.1897150 (PMC13618056; doi:10.3389/fvets.2026.1897150)
Supplement: Supplementary file 5 [file Table_2.docx]

**Supplementary Table S2.**

**Metadata of ITS/rDNA sequences used for phylogenetic analysis**

| **No.** | **Species name in tree** | **Strain/isolate label** | **GenBank accession number** | **Host/source** | **Country/region** | **Note** |
| --- | --- | --- | --- | --- | --- | --- |
| 1 | *Trichophyton verrucosum* | CCF 4611 | LN614523.1 | Back skin of 11-year-old girl; host: *Homo sapiens sapiens* | Czech Republic: Cervena Voda | Reference sequence |
| 2 | *Trichophyton verrucosum* | DSM 112239 | OM326338.1 | Skin scales, tinea corporis, female, 56 years; host: *Homo sapiens* | Germany | Reference sequence |
| 3 | *Trichophyton verrucosum* | CCF 4614 | LN614522.1 | Skin of the shoulder and back of 6-year-old boy; host: *Homo sapiens sapiens* | Czech Republic: Pardubice | Reference sequence |
| 4 | *Trichophyton verrucosum*-related isolate | KZ_Tr1 | PZ441530 | Cattle | Kazakhstan, Almaty region | This study |
| 5 | *Trichophyton verrucosum* | CBS 142192 | KY623476.1 | Right arm of 3-year-old boy | China: Qianxi, Guizhou | Reference sequence |
| 6 | *Trichophyton verrucosum*-related isolate | KZ_Tr2 | PZ441531 | Cattle | Kazakhstan, Almaty region | This study |
| 7 | *Trichophyton verrucosum* | 1482 | MN295947.1 | Not specified in GenBank screenshot | Not specified in GenBank screenshot | Reference sequence |
| 8 | *Trichophyton benhamiae* | UKJ 488/23 | PV768555.1 | Neckline of 37-year-old female; contact with guinea pig | Germany: Jena | Reference sequence |
| 9 | *Trichophyton erinacei* | ATCC 28446 | EF631615.1 | Not specified in GenBank screenshot | Not specified in GenBank screenshot | Reference sequence |
| 10 | *Trichophyton violaceum* | BMU02087 | EF631624.1 | Not specified in GenBank screenshot | Not specified in GenBank screenshot | Reference sequence |
| 11 | *Trichophyton rubrum* | 1600 | MN295946.1 | Not specified in GenBank screenshot | Not specified in GenBank screenshot | Reference sequence |
| 12 | *Trichophyton rubrum* | TWCC 57922 | LC369522.1 | Wound pus from a subcutaneous nodule; host: *Homo sapiens* | Japan | Reference sequence |
| 13 | *Trichophyton rubrum* | BMU01672 | EF631619.1 | Not specified in GenBank screenshot | Not specified in GenBank screenshot | Reference sequence |
| 14 | *Trichophyton schoenleinii* | ATCC 22775 | EF631620.1 | Not specified in GenBank screenshot | Not specified in GenBank screenshot | Reference sequence |
| 15 | *Trichophyton tonsurans* | 419 | MN295953.1 | Not specified in GenBank screenshot | Not specified in GenBank screenshot | Reference sequence |
| 16 | *Trichophyton tonsurans* | 1645 | MN295945.1 | Not specified in GenBank screenshot | Not specified in GenBank screenshot | Reference sequence |
| 17 | *Trichophyton tonsurans* | 125 | MN295962.1 | Not specified in GenBank screenshot | Not specified in GenBank screenshot | Reference sequence |
| 18 | *Trichophyton verrucosum* | BMU03357 | EF631623.1 | Not specified in GenBank screenshot | Not specified in GenBank screenshot | Reference sequence; positioned outside the main *T. verrucosum*-related cluster |
| 19 | *Trichophyton mentagrophytes* | UKJ 594/19 | MN064822.1 | Infected human scales; host: *Homo sapiens* | Germany | Reference sequence |
| 20 | *Trichophyton mentagrophytes* | 28 | MN295960.1 | Not specified in GenBank screenshot | Not specified in GenBank screenshot | Reference sequence |
| 21 | *Trichophyton mentagrophytes* | BMU03104 | EF631618.1 | Not specified in GenBank screenshot | Not specified in GenBank screenshot | Reference sequence |
| 22 | *Arthroderma vanbreuseghemii* | IFM 41271 | AB458202.1 | Host: *Homo sapiens* | Finland | Related dermatophyte reference; anamorph: *Trichophyton mentagrophytes* |
| 23 | *Trichophyton mentagrophytes* | 309 | MN295955.1 | Not specified in GenBank screenshot | Not specified in GenBank screenshot | Reference sequence |
| 24 | *Trichophyton mentagrophytes* | 129 | MN295961.1 | Not specified in GenBank screenshot | Not specified in GenBank screenshot | Reference sequence |
| 25 | *Trichophyton mentagrophytes* | 2024-N157 | PV185768.1 | Skin scale of patient with dermatophytosis; host: *Homo sapiens* | Viet Nam: Hanoi | Reference sequence |
| 26 | *Microsporum canis* | ATCC MYA-4605 | GU291265.1 | Not specified in GenBank screenshot | Not specified in GenBank screenshot | Outgroup |

**Note:** Metadata were extracted from the GenBank corresponding to the ITS/rDNA sequences used in the phylogenetic tree. When host, isolation source, or geographic origin was not visible in the GenBank, the field was recorded as “Not specified in GenBank”. KZ_Tr1 and KZ_Tr2 were generated in the present study and are listed as cattle-associated *T. verrucosum*-related isolates from Kazakhstan.
